# Ahead of the (ROC) Curve: A Statistical Approach to Utilizing Ex-Gaussian Parameters of Reaction Time in Diagnosing ADHD Across Three Developmental Periods

**DOI:** 10.1017/S1355617721000990

**Published:** 2021-09-07

**Authors:** Hilary Galloway-Long, Cynthia Huang-Pollock, Kristina Neely

**Affiliations:** 1 Department of Psychology, The Pennsylvania State University, University Park, PA, USA; 2 VA Puget Sound Healthcare System, American Lake Division, WA, USA; 3 School of Kinesiology, Auburn University, Auburn, AL, USA

**Keywords:** ADHD, Reaction time, Processing speed, Diagnostic discriminability, Receiver operating characteristic curve, Inhibitory control

## Abstract

**Introduction:**

Performance on executive function (EF) tasks is only modestly predictive of a diagnosis of Attention Deficit Hyperactivity Disorder (ADHD), despite the common assumption that EF deficits are ubiquitous to the disorder. The current study sought to determine whether ex-Gaussian parameters of simple reaction time are better able to discriminate between children and adults with and without ADHD, compared with traditional measures of inhibitory control.

**Methods:**

Receiver Operating Characteristic (ROC) analyses and the area under the curve (AUC) were used to examine the ability of performance on two commonly used tasks of inhibitory control (i.e. stop signal reaction time (SSRT) and go-no-go tasks) to predict ADHD status in preschool (*N* = 108), middle childhood (*N* = 309), and young adulthood (*N* = 133).

**Results:**

Across all samples, SSRT, go-no-go percentage of failed inhibits, and standard deviation of reaction (SDRT) time to “go” trials, all successfully discriminated between individuals with and without ADHD. Ex-Gaussian decomposition of the RT distribution indicated that both larger tau and larger sigma drove findings for SDRT. Contrary to predictions, traditional measures of inhibitory control were equal if not better predictors of ADHD status than ex-Gaussian parameters.

**Conclusions:**

Findings support ongoing work to quantify the separate contributions of cognitive subprocesses that drive task performance, which in turn is critical to developing and improving process-based approaches in clinical assessment.

## INTRODUCTION

Attention Deficit Hyperactivity Disorder (ADHD) is a neurodevelopmental disorder characterized by difficulties sustaining attention, hyperactivity, and impulsivity (American Psychiatric Association, 2013). Much of the modern literature that has repeatedly documented the presence of executive function (EF) deficits in children and adults with ADHD has done so by demonstrating high error rates and slower/more variable response times (RT) on tasks commonly used to index executive control (e.g. go-no-go, continuous performance tasks, complex memory span, Stroop, etc.). However, group effect sizes remain modest, and there is substantial overlap in performance distributions among those with and without ADHD (Epstein et al., 2011; Kofler et al., 2013; Lijffijt, Kenemans, Verbaten, & van Engeland, 2005; Nigg, Willcutt, Doyle, & Sonuga-Barke, 2005). In fact, mean performance on reaction time and traditional paper-and-pencil neuropsychological tasks are only weakly predictive of diagnostic status in children or adults. AUC values in those studies have ranged from .56 to .72, with .50 indicating chance prediction (Doyle et al., 2005; Edwards et al., 2007; Emser et al., 2018; Faraone et al., 2016; Teicher, Polcari, Fourligas, Vitaliano, & Navalta, 2012).

Although genuine cognitive heterogeneity within the broad phenotype of ADHD likely exists (Fair, Bathula, Nikolas, & Nigg, 2012; Wahlstedt, Thorell, & Bohlin, 2009), it may also be that the tendency to rely on indices of central tendency (e.g. mean, standard deviation, median) does not provide the best descriptors of performance and subsequently prevents the identification of what could be more widespread cognitive dysfunction within the ADHD population. To be specific, RT distributions are not normal; they are positively skewed. Bound by a fastest possible response time of 0 milliseconds and potentially infinitely slow responses, they are therefore best represented by the non-normal ex-Gaussian distribution (Dawson, 1988; Van Zandt & Townsend, 2014). The ex-Gaussian distribution integrates an exponential distribution with a normal distribution to account for the positive skew. The parameters mu and sigma refer to the mean and standard deviation of the normal portion of the distribution, respectively; tau characterizes the mean of the exponentially shaped (skewed) right-sided tail portion of the distribution (Lacouture & Cousineau, 2008).

There has in fact been some evidence that an approach that separates the RT distribution better distinguishes children with and without ADHD compared with standard indices of performance. For example, significant ADHD group effects have been reported for tau (but not mean RT) across a range of reaction time tasks in middle childhood (Epstein et al., 2011). In addition, an early study in school-aged boys with ADHD found that whereas *tau* and standard deviation of RT were highly diagnostic of ADHD (both AUC = .96), *mu* and *sigma* held much lower diagnostic value (both AUC = .62) (Leth-Steensen, Elbaz, & Douglas, 2000).

Like the ex-Gaussian parameters, alternative mathematical methods such as the diffusion model (Ratcliff & McKoon, 2008; Ratcliff, Smith, Brown, & McKoon, 2016; A. Voss, Nagler, & Lerche, 2013) and linear ballistic accumulation model (Brown & Heathcote, 2008) are also capable of more accurately describing performance than traditional neuropsychological indices of performance. In the last 10 years, their use has also gained significant traction in the clinical and developmental research literatures (Huang-Pollock et al., 2020; Ratcliff, Love, Thompson, & Opfer, 2012; White, Ratcliff, Vasey, & McKoon, 2010). However, unlike ex-Gaussian parameters, the calculation of Diffusion Modeling (DM) and Linear Ballistic Accumulation (LBA) parameters is not straightforward, which creates a significant barrier for adoption for clinical use. In contrast, ex-Gaussian parameters are easily calculated and, if they demonstrate predictive utility in distinguishing among children and adults with ADHD, would provide an easy-to-implement clinical tool for evaluative purposes.

Using ROC curves and the AUC, the current study evaluates the degree to which ex-Gaussian parameters of reaction time might better discriminate between children and young adults with and without ADHD, compared with traditional methods of indexing cognition. ROC analyses are a natural model for how clinical decisions are made and how data are used in evaluative processes (Youngstrom, 2014). In this process, a categorical diagnosis (i.e. ADHD *vs.* non-ADHD) is predicted by data obtained during the course of an evaluation. Due to their ability to efficiently quantify the sensitivity and specificity of a decision-making procedure, and their ability to allow for the direct comparison of the predictive utility of different tests, ROC/AUC analyses are now widely taught in graduate assessment courses to aide in clinical decision-making (Haynes, O’Brien, & Kaholokula, 2011; Youngstrom, Prinstein, Mash, & Barkley, 2020).

Based on previous work finding large ADHD-related effects for intraindividual variability, we hypothesize that SDRT, tau, and sigma will outperform indices of central tendency (i.e. mean RT and mu) in the prediction of ADHD. We further predict that tau will outperform SDRT and sigma. However, disinhibition is central to mechanistic theories of ADHD (Barkley, 1997; Nigg, 2001; Slaats-Willemse, Swaab-Barneveld, de Sonneville, van der Meulen, & Buitelaar, 2003; Wright, Lipszyc, Dupuis, Thayapararajah, & Schachar, 2014). Thus, the strongest test of clinical utility would be to directly compare tau’s discriminability against indices of inhibitory control (i.e. percent failed inhibits on a go-no-go task or the SSRT on a SSRT task). That being said, intraindividual variability has long been known to normatively decrease from childhood through young adulthood (Tamnes, Fjell, Westlye, Ostby, & Walhovd, 2012; Williams, Hultsch, Strauss, Hunter, & Tannock, 2005). In the current study, we therefore also predict that tau’s predictive utility will be the strongest in older *versus* younger participants.

## METHODS

### Participants and Procedures

Data for this study was collected within larger programs of research aimed to better understand the cognitive mechanisms that contribute to the execution of complex behaviors, including those associated with psychopathology and ADHD. Three separate samples were recruited for the study: “preschool” (5–6 years) (Karalunas, Bierman, & Huang-Pollock, 2020), “school-aged” (8–12 years) (Huang-Pollock et al., 2017; Karalunas & Huang-Pollock, 2013), and “young adult” (18–25 years) (Neely et al., 2017). Prior to participating, verbal assent from children was obtained. Informed written consent was obtained from parents of child participants and adult participants. In the school-aged and adult samples, parents/adults were monetarily compensated for their participation ($100 for parents, $40 for adults). In the preschool and school-aged samples, teachers were also compensated ($10) for their time completing behavioral rating scales.

#### Exclusions

Children were excluded if parents reported any sensorimotor disability, frank neurological disorder (e.g. seizures), autism, psychosis, or if limited proficiency in English would impair full participation. Adults were excluded for the abovementioned reasons, as well as for a history of concussion that resulted in a loss of consciousness for more than 10 min.

Participants with low estimated Full-Scale IQ were excluded from all studies (i.e. >70 on a two-subtest short form comprising Vocabulary and Matrices from the Stanford Binet −5 in the preschool sample; >80 on a two-subtest short form comprising Vocabulary and Matrices of the WISC-IV (school-aged) or WAIS-IV (adult) samples). Other common psychiatric disorders, including anxiety, depression, oppositional defiant disorder, and conduct disorder, were assessed using parental report on the DISC-IV or self-report using the Adult Self-Report Scale (Achenbach & Rescorla, 2003), but were not exclusionary.

#### Medication use

All participants prescribed a stimulant medication were asked to discontinue their use 24–48 h in advance (*N*_preschool_ = 1, *N*_school aged_ = 83, *N*_young adult_ = 42). Participants prescribed a non-stimulant medication were excluded from participation. However, one preschool participant was prescribed a non-stimulant medication (Strattera). Because it has a longer half-life, the child did not discontinue medication use.

### Diagnostic Determination

Table 1 provides descriptive statistics for demographic and diagnostic information within each sample alongside results from relevant F- and Chi-square tests comparing groups.

#### Child samples

Children were recruited from the Centre, York, and Harrisburg counties of Pennsylvania. Children were identified as having ADHD if they met full DSM criteria for a diagnosis of ADHD on the Diagnostic Interview Schedule for Children version IV (DISC-IV) (Shaffer, Fisher, Fucas, Dulcan, & Schwab-Stone, 2000), including symptom count, chronicity, impairment, and cross-situational severity. In the preschool sample, this process resulted in an *N* = 75 (23 girls) identified as ADHD. In the school-aged sample, this resulted in an *N* = 216 (73 girls) identified as ADHD.

Following DSM field trials, final symptom count was derived following the “or” algorithm to integrate parent and teacher report: a symptom was counted as present if either the parent (on the DISC-IV) or the teacher (on the ADHD-RS) indicated that a symptom was present “often” or “very often” (Lahey et al., 1994). As evidence of cross-situational severity, on standardized behavior rating forms, at least one parent *and* one teacher report were required (a) to exceed a *t*-score of 61 (85th percentile) on ADHD-related indices of the BASC (Hyperactivity or Inattention) or Conner’s (Hyperactivity, Inattention, or ADHD Index), or (b) have endorsed ≥ three inattentive or three hyperactive/impulsive symptoms, or ≥ four total symptoms, as *often* or *very often* on the ADHD Rating Scale.

Children were classified as non-ADHD control participants if they had never been diagnosed with or treated for ADHD in the past, parent and teacher reports on all above listed rating scales were below the 80th percentile (*t*-score ≤ 58), and <3 inattentive symptoms and <3 hyperactive/impulsive symptoms, and <4 total symptoms using the “or” algorithm. In the preschool sample, this resulted in an *N* = 33 (15 girls). In the school-aged sample, this resulted in an *N* = 93 (46 girls).

#### Adult sample

Adults were identified as having ADHD (*N* = 62, 35 women) if they met full DSM criteria for a diagnosis of ADHD in adulthood. Following DSM, at least five symptoms of inattention or hyperactivity/impulsivity were required to be present. Cross-situational severity and impairment were determined by self-report on the Conners’ Adult ADHD Diagnostic Interview (CAADID) (Conners, Epstein, & Johnson, 2001), Connors’ Adult ADHD Rating Scales (CAARS), and the Achenbach Adult Self-Report (ASR) (Achenbach & Rescorla 2003).

Adults were classified as non-ADHD control participants (*N* = 72, 30 women) if they had never been diagnosed with or treated for ADHD in the past and reported <2 symptoms of inattention or hyperactivity/impulsivity and <3 total symptoms on the CAADID.

#### Diagnostic validity

Validating the diagnostic strategy described above, there were significant differences in the number of inattentive and hyperactive symptoms across groups (all *p* < .001, and *η*^2^ > .2; See Table 1). Consistent with expectations (e.g. Martel, 2013), there were more males than females with ADHD, compared with typically developing controls. Diagnostic groups did not differ in age (all *F* < .82, *p* > .37, *η*^2^ < .001). Though non-ADHD controls had higher mean FSIQ than same-aged ADHD peers in both the preschool and adult samples (both *p* < .001, both *η*^2^ > .11), they were well within the population average.

#### Cognitive tasks

**Fig. 1.**
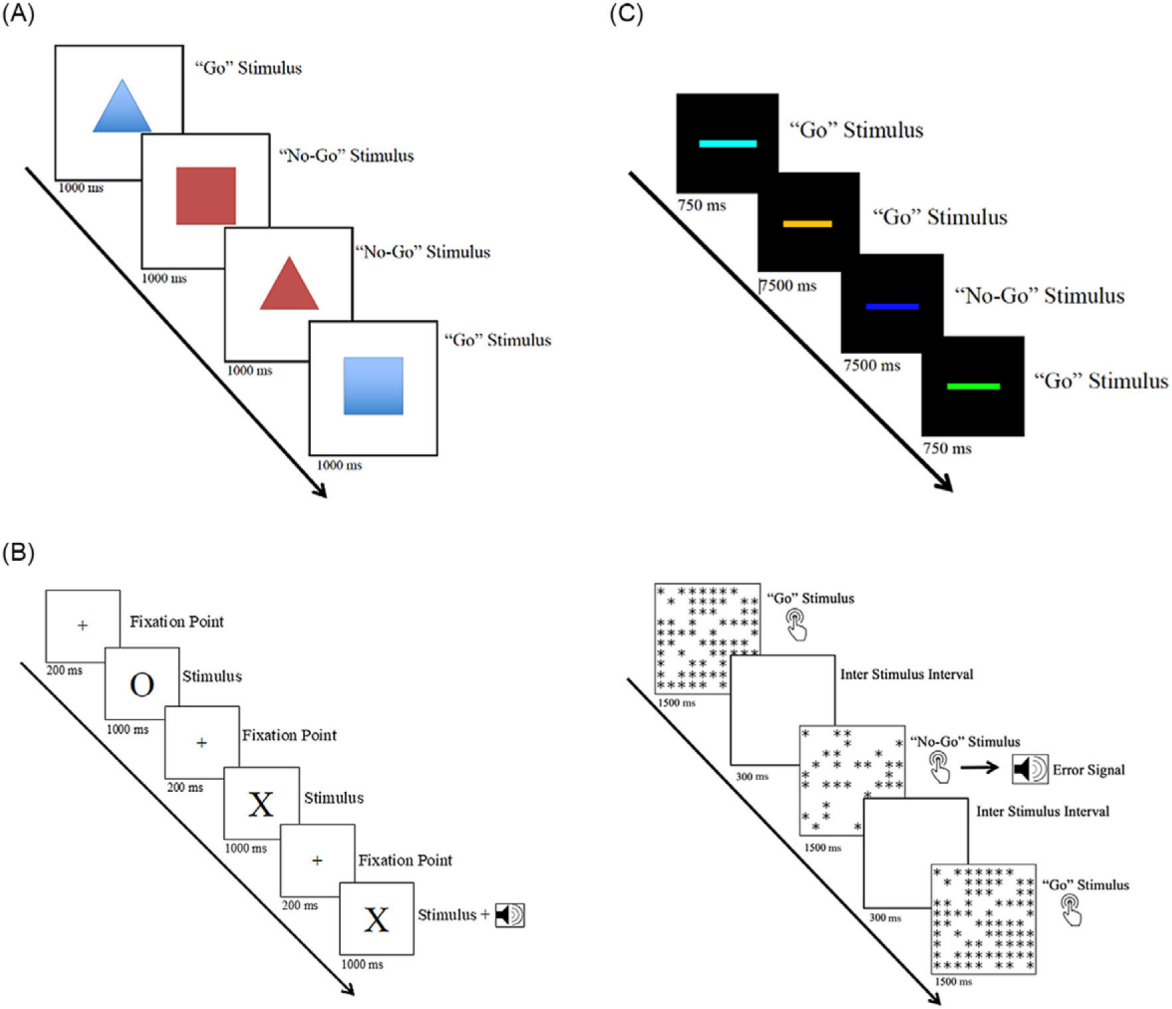
Illustration of the Go/No-Go task used in the preschool sample. (B) Illustration of the Stop Signal Reaction Time (SSRT) task and Go-No-Go tasks used in the school-aged sample. (C) Illustration of the Go/No-Go task used in adult sample.

Figure 1 provides an illustration for all tasks. Parameter specifications were chosen to establish a rapid, prepotent response that must be inhibited similar to other inhibitory tasks used in both clinical practice and research (Wright et al., 2014).

#### Preschool go/no-go task

Over 60 trials, children consecutively viewed one of four shapes (blue triangle, blue square, red triangle, and red square). They were asked to make a key press when they saw a blue shape (“go” trial), but to withhold a response when they saw a red shape (“no-go” trial). Each shape appeared for 1000 ms, and children were given 2000 ms to respond. The next trial began immediately after a response or after 2000 ms had elapsed. A brief error tone was provided after incorrect responses. Of the trials, 70% (*n* = 42 trials) were Go trials and 30% (*n* = 18 trials) were No-Go. Time to completion was approximately 6 min, including task instructions.

#### School-aged go/no-go task

Ten blocks of 80 trials were administered with optional rest periods in between. At the start of each trial, a number of white asterisks filled random positions in a 10 × 10 array centered on a black screen (Huang-Pollock et al., 2017). Children were told “We’re going to play a game called the Candy Factory now. Some of the boxes of candy that the factory makes have a lot of candy in them, and some only have a little. But, the sorter is broken! We need your help! Every time you see a box that has ‘a lot’ of candy, press the spacebar. Don’t press anything if the box has ‘a little’ bit of candy. This is a hard game but try to work as quickly as you can without making mistakes. Let’s try some for practice.” Four practice trials were then given. Stimuli remained onscreen for 1500 ms during which responses could be collected. The next trial began 300 ms after a response or after the 1500 ms had elapsed. Of the trials, 75% were “go” trials (*n* = 600 trials, stimuli selected at random without replacement) and contained 61–70 asterisks. The remaining 25% were “no-go” trials (*n* = 200 trials) and contained 31–40 asterisks. Children were not provided any instruction on how to distinguish “a lot” from “a little,” but were provided with a brief error tone after incorrect responses. Time to completion was approximately 20 min, including task instructions.

#### School-aged stop signal reaction time task

Children were administered a 200-trial tracking version of the Logan Stopping Task that ensures a 50% failed inhibit rate for all participants (Logan, 1985). Five blocks of 40 trials were administered with optional rest periods in between. At the start of each trial, a central fixation point appeared for 200 ms. An “X” or an “O” next appeared on screen for 1000 ms, and children were the given 2300 ms to indicate with a keystroke which letter had appeared. The next trial onset was immediately after a response or after 2300 ms had elapsed. Of the trials, 75% were “go” trials (*n* = 150). The other 25% (*n* = 50) were “stop” trials in which an auditory tone was presented to indicate that children should not respond. Time to completion was approximately 20 min, including task instructions.

An initial mean reaction time (MRT) was determined based on 20 practice “go” trials presented prior to the start of the experimental blocks. The auditory stop tone was initially set to occur 250 ms before the MRT. The MRT was then dynamically recalculated after each correct go trial, and the delay at which the stop tone was presented was adjusted dynamically in 50 ms increments to maintain an overall ~50% accuracy rate^1^. SSRT is defined as the amount of time a child needs to successfully inhibit a response 50% of the time. It was calculated by subtracting the mean stop signal delay from the child’s MRT.

#### Adult go/no-go task

Four blocks of 100 trials were administered, with 12.5 s of rest between each block. At the start of each trial, a central fixation point (a horizontal white bar) appeared in the center of the screen for 500 ms against a black background. The bar then turned green, aqua, orange, yellow, or blue. Participants were asked to make a key press as quickly as possible (“go” trials) except if the bar was blue (“no go” trial). Stimuli appeared onscreen for 750 ms during which responses could be collected. The next trial began after the 750 ms had elapsed. Of the trials, 75% (*n* = 300 trials) were “go” and 25% (*n* = 100) were “no go.” No feedback concerning accuracy was provided. Time to completion was approximately 15 min, including task instructions.

#### Calculating reaction time to “Go.”

For the SSRT and GNG tasks, mean and standard deviation of reaction time were calculated using all correct “go” responses, not preceded by a “no-go” trial to reduce the possible effect of inhibitory control on the RT distribution (Schachar et al., 2004). Additionally, trials < 150 milliseconds were excluded as they are generally thought to represent anticipatory error. Given the ease of each task, rates of excluded trials were exceptionally small. Specifically, 1.6% of trials were removed for preschool GNG task, 1.3% of trials for the school-aged GNG task, .58% of trials for the school-aged SSRT task, and .19% of trials for the adult GNG task.

The normality of reaction time distributions for each task was assessed using traditional data exploration techniques. Ex-Gaussian parameters mu, sigma, and tau were computed using an egfit tool in MATLAB (Lacouture & Cousineau, 2008), using the same RT trial criteria described for mean and standard deviation. This function performs an iterative search process to compare the observed RT distribution with an ex-Gaussian probability density function (PDF) using a Simplex method. In each iteration, the parameter values of the PDF are adjusted until maximum fit to the observed data is achieved. To validate that the ex-Gaussian distribution is an appropriate fit for the data, a simulation-recovery study was conducted using the “rexgauss()” function from the R package “retimes.” This generates simulated response time data based on the ex-Gaussian parameters derived from the empirical response times, which is then fit to the ex-Gaussian distribution using the same procedures described above.

For the school-aged and adult samples, correlations between the simulated and empirical values for mu, sigma, and tau were all above .8, indicating that the parameters fit well. For the preschool sample, the correlation between simulated and empirical mu was above .8. Sigma and tau were both low (.4), indicating poor fit, likely due to the relatively low number of trials. Due to strong fit for mu and sigma, we proceded with analyses for the preschool sample but address the implications of uncertain fit for tau in that sample in the Discussion.

#### Data analytic plan

For all tasks, dependent variables include: mean and standard deviation as well as ex-Gaussian parameters (mu, sigma, and tau) for “go” trials. In the SSRT task, SSRT was also a dependent variable. ROC analyses are commonly used to evaluate the sensitivity and specificity of test predictions (Hajian-Tilaki, 2013, 2018; Hajian-Tilaki, Hanley, & Nassiri, 2011; Hanley, 1989; Hanley & McNeil, 1983; Youngstrom, 2014). ROC analyzes graph sensitivity on the y-axis (values ranging from 0 to 1), and false alarm rate (e.g. the inverse of specificity or, 1-specificity) on the x-axis (values also ranging from 0 to 1). Curves farthest to the top left of the graph are therefore both highly sensitive and specific, while those closest to the diagonal line are neither and perform close to chance. The AUC statistic quantifies each curve’s distance from chance. AUC values range from 0 to 1, with 1 indicating perfect sensitivity and specificity, .5 accuracy equal to chance, and 0 complete failure.

## RESULTS

### Diagnostic Group Differences

Table 2 provides descriptive statistics for performance on the SSRT task and GNG task by diagnostic group and for all samples. Results did not change when analyses were restricted by gender, but are reported in the Supplemental Tables 1 and 2 for interested readers. Main effects for ADHD status were observed; children/adults with ADHD had more failed inhibits on the GNG tasks in all samples, and school-aged children had longer SSRT (all *p* < .05, all *η*^2^ > .046). There was no main effect of ADHD status for any sample in MRT to “go” trials, but SDRT was larger among children/adults with ADHD in all tasks and all samples (all *p* < .01, all *η*^2^ > .048). Examination of the ex-Gaussian parameters found sigma and tau to be significantly longer in the school-aged sample (all *p* < .03, all *η*^2^ > .02); tau was also longer in the young adult sample (*p* = .001, *η*^2^ = .085).

#### Determining discriminability

**Fig. 2.**
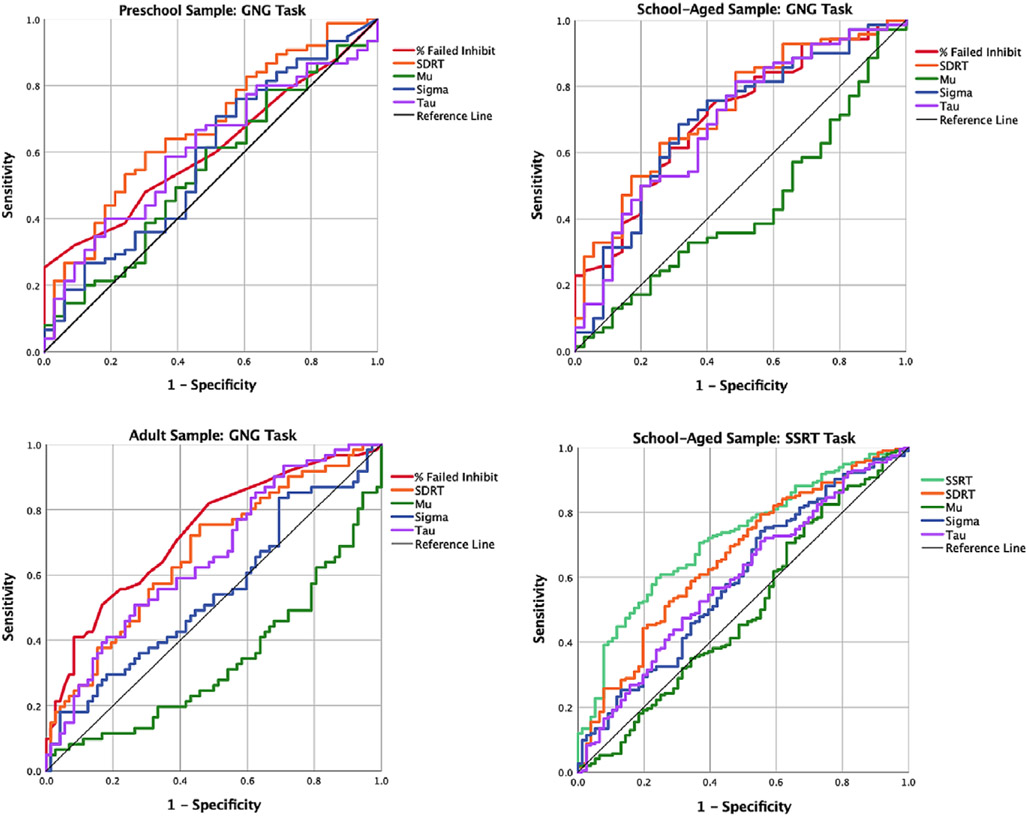
ROC curves for all tasks, distinguishing children with ADHD from typically developing peers.

The diagnostic utility of each task parameter was assessed using ROC curves and evaluating the AUC (see Table 3; Figure 2).

As expected, given their history in the study of ADHD, SSRT and % failed inhibits on a GNG task discriminated between ADHD and typically developing participants in both the school and adult samples (all AUC > .71, all *p* < .001). Percent failed inhibits in the preschool sample predicted ADHD in the correct direction, but was not significant (AUC = .608, *p* = .076).

Of the performance parameters formed by simple reaction time to “go” trials, SDRT discriminated between diagnostic groups in all tasks and all samples (all AUC > .659, all *p* < .005). Ex-Gaussian parameters allow for the decomposition of the RT distribution to illuminate the source of this effect. In the school-aged sample, the ability of SDRT to do so was driven by both sigma and tau (all AUC > .592, all *p* < .02); in the young adult sample, it was driven by tau (AUC = .655, *p* = .002). Sigma also discriminated between groups in the school-aged sample for both tasks (both AUC > .592, both *p* < .02). Decomposition of the RT distribution did not provide any additional information for the preschool sample.

#### Comparing strength of predictive utility

We next compared the diagnostic discriminability of the most consistent performance parameters (i.e. SSRT, percent failed inhibits, SDRT, sigma, and tau). Z-scores representing differences in the strength of discriminability between any two variables were generated. Z-scores were created by using their AUC values, standard errors and the correlations between the two predictors within each group (Hanley & McNeil, 1983) (See Table 4). For the school-aged sample, both SSRT and SDRT predicted ADHD status better than tau, and percent failed inhibits predicted ADHD better than sigma. Among the adult sample, percent failed inhibits also outperformed sigma. All other comparisons were equally predictive of ADHD, with none being stronger than the other.

## DISCUSSION

Preschool and school-aged children, as well as adults with ADHD, had slower stop signal reaction times (derived from the SSRT task), a greater percentage of failed inhibits (derived from a go-no-go task), and larger SDRT to “go” trials than those without ADHD. Ex-Gaussian decomposition of the RT distributions indicated that the findings for SDRT were driven by both sigma and tau in school-aged group and by tau in the adult sample.

The health of multiple broad subprocesses necessary for successful task performance (e.g. perceptual encoding, decision-making, and fine-motor output) can be inferred from the shape of the RT distribution (Luce, 1986; Myerson, Hale, Zheng, Jenkins, & Widaman, 2003; Rotello & Zeng, 2008; Smith & Ratcliff, 2004). Competing theories that associate each ex-Gaussian parameter with a particular psychological construct have been proposed (see: Matzke & Wagenmakers, 2009 for a review of the literature). One particularly well-received and common argument suggests that because motor preparation/execution and informational encoding are relatively automatic functions, the general speed or efficiency of these processes are normally distributed and are best measured by indices of central tendency (i.e. mu and sigma). In contrast the more effortful or attentional processes are best described by the exponential tail of the distribution (i.e. tau) (Abney, McBride, & Petrella, 2013; Balota & Spieler, 1999; Gmehlin et al., 2014; Gordon & Carson, 1990; Hockley, 1984; Luce, 1986; Madden et al., 1999; Moret-Tatay et al., 2016; Rotello & Zeng, 2008).

The specific conceptual interpretation of tau adopted here is that tau represents the speed of information accumulation during the decision-making. This interpretation is strongly informed by a mathematical model of choice reaction time task performance known as the diffusion model (Ratcliff, 2002, 2014; Andreas Voss, Rothermund, & Voss, 2004). Though not a perfect 1:1 association, “drift rate,” a parameter from the diffusion model that indexes the speed of information or evidence accumulation during decision-making, is substantively negatively correlated with tau (Karalunas & Huang-Pollock, 2013; Matzke & Wagenmakers, 2009). The group effects reported herein have been replicated extensively in the literature and add to the growing correlational as well as experimental evidence suggesting that slower rate of evidence accumulation helps to explain why ADHD is associated with poor performance on many tasks of EF (Karalunas & Huang-Pollock, 2013; Weigard & Huang-Pollock, 2017; Weigard, Huang-Pollock, Brown, & Heathcote, 2018).

Findings for tau were not seen in the preschool sample, however. Preschool children with ADHD did not have longer taus than their non-ADHD counterparts. The lack of an ADHD-related group effect in this youngest group is likely representative of sample-specific methodological differences, rather than true developmental differences. Because of its influence on cognitive activation, the interstimulus interval is commonly used to experimentally manipulate the shape of RT distributions. RTs are slower and SDRTs are larger when interstimulus intervals are longer in typically developing children and children with ADHD (Andreou et al., 2007; Epstein et al., 2011; Huang-Pollock et al., 2017; Sanders, 1970; Sergeant, 2000; Wiersema, van der Meere, Roeyers, Van Coster, & Baeyens, 2006). In the current study, the interstimulus interval for the preschool GNG task was 2000 ms and may not have been long enough to elicit the ADHD-related atypically longer tail. A recent study of ex-Gaussian parameters of reaction time in preschool students at risk for ADHD found that sigma and tau were greater in children with ADHD than typically developing controls when using an interstimulus interval of 3000 ms, but not with an interstimulus interval of 1500 ms (Hwang-Gu et al., 2019).

Because data are best fit to a distributional model when there are many individual data points (Lacouture & Cousineau, 2008), it is also possible that the smaller number of trials in the preschool GNG task (*n* = 60 total trials, 42 go-trials) influenced findings. Parameter recovery for mu, sigma, and tau for school-aged and young adults, as well as mu for the preschool sample, was all quite strong (all *r* > .80). However, the sigma and tau parameters for the preschool sample produced a simulated RT distribution that was significantly correlated with the empirical distribution (*r* = .4), but still below the commonly applied threshold (*r* = .8). Therefore, the lack of group effects for preschool tau could also be due to lower trial numbers producing less accurate estimates of tau, as opposed to suggesting that RT variability functions differently in young children with ADHD. Hwang-Gu et al. (2019)’ s GNG task had 200 total trials, which would be expected to yield better fits, although a parameter recovery study was not performed in that study, and fit was not reported. Future studies seeking to replicate this work in preschool-aged children would need to carefully balance task length needed to maintain adequate motivation throughout task administration, with enough trials to ensure strong fit for the tail of the distribution.

SSRT, % failed inhibit, SDRT, tau, and sigma (for the school-aged sample) all predicted the presence of ADHD. However, in direct head-to-head comparisons, SSRT was a much stronger predictor of ADHD status than any other metric, contrary to hypotheses. Furthermore, despite being a more specific index of variability at the tail of the distribution, tau did not outperform SDRT. That being said, each parameter independently predicted ADHD status to a degree that was equal to or stronger than those reported in studies of similar tasks that are currently and commonly used in clinical practice. For example, studies of the Conners’ CPT-II have reported AUC values for standard error ranging between .63 and .71, and for percentage of failed inhibits ranging between .43 and .67 (Jarrett, Meter, Youngstrom, Hilton, & Ollendick, 2018; Teicher et al., 2012). Of particular note were the predictive strengths of the both the SSRT in the school-aged sample (AUC = .717) and percentage if failed inhibits in the adult sample (AUC = .730). While go-no-go tasks are not uncommon in clinical evaluations (e.g. Connor’s CPT), the SSRT is not often used, despite being one of the most commonly employed tasks to evaluate cognitive performance in ADHD within research settings (Crosbie et al., 2013; Fipszyc & Schachar, 2010; Nigg et al., 2018). These results suggest that performance on a SSRT task may be another useful tool in the evaluation of ADHD within clinical settings. However, recent work finds that the size of the group effect could be attenuated if an integration approach (as opposed to another commonly used approach, the mean approach, used in the current study) is used to calculate the SSRT (Verbruggen, Chambers, & Logan, 2013). Thus, it remains possible, although unlikely, that if the SSRT had been designed using an integration approach, its predictive utility would be reduced.

Because they are designed to reflect the signs and symptoms of disorder, AUC values for behavioral rating scales in the prediction of disorder are commonly quite large (ranging from .70s to .90s) due to that tautological advantage (Chen, Faraone, Biederman, & Tsuang, 1994; de la Osa, Granero, Trepat, Domenech, & Ezpeleta, 2016; Hudziak, Copeland, Stanger, & Wadsworth, 2004; Lampert, Polanczyk, Tramontina, Mardini, & Rohde, 2004; Raiker et al., 2017). Performance on cognitive tasks lacks this tautological advantage in predicting disorder, but the value in their continued study is their potential to speak to the possible causal and transdiagnostic mechanisms that may contribute to the development or maintenance of disorder (Devena & Watkins, 2012; Doyle et al., 2005; Jarrett et al., 2018; Pineda, Puerta, Aguirre, García-Barrera, & Kamphaus, 2007; Teicher et al., 2012).

Effective screening tools are those that can be rapidly and inexpensively administered. They are designed to select a relatively low optimal cutoff to maximize the number of individuals warranting further evaluation, while tools used for ruling out a diagnosis tend to use higher cutoffs. Possible consequences of a “false positive” diagnosis of ADHD include social stigma associated with diagnosis, failure to make an appropriate alternative diagnosis, or even possible iatrogenic effects of an unnecessary medication (Abramovitch, 2016; Birnbaum et al., 2005; Evans, Morrill, & Parente, 2010; Wiener et al., 2012). Possible consequences of a “false negative” diagnosis could include the downstream effects of chronic untreated ADHD including academic underachievement/school failure, increased peer and familial conflict, dangerous or sensation-seeking behavior, or illegal drug use (Biederman et al., 2004; Birnbaum et al., 2005; Eakin et al., 2004; Goodman, 2007; Harpin, 2005; Minde et al., 2003).

Cognitive tasks such as those within the current study are likely not suited to functioning as screeners as they cannot be quickly and cheaply administered. Additionally, because a large portion of people meeting diagnostic criteria do not show impairments on cognitive tasks such as these, their use as a method to rule out a diagnosis would likely result in many missed diagnoses. The current work is consistent with process-based approaches in clinical assessment and is aligned with current efforts to develop and improve upon a new generation of neuropsychological tests to evaluate cognitive weaknesses and treatment response (Au, Piers, & Devine, 2017; Bornstein, 2011).

### Limitations and Future Directions

While the same broad go-no-go paradigm was implemented with all three age groups, the specific tasks administered did vary in several ways, including type and number of stimuli, complexity, time between stimuli, and feedback regarding accuracy. Each task was designed to be developmentally appropriate in length and motivational demand requirements, but those design decisions also introduced sample specific confounds that may have led to variation in performance, rather than true age-related differences. In the preschool-aged task, specifically, the decision to design the task with fewer trials may have contributed to the lack of findings in that age group. It is recommended that future work continue to develop paradigms that are able to maintain motivation and active assent during participation, while also ensuring an adequate number of trials to ensure strong parameter recovery across age groups, as demonstrated by a parameter recovery study to ensure good fit to the data. Furthermore, future studies should aim for a more even gender balance, enabling careful evaluation of if and how male and female participants demonstrate genuinely different cognitive performance profiles, requiring separate normative values.

### Summary and Conclusions

Across multiple developmental periods, traditional indices of inhibitory control as well as variance in the speed to “go” all successfully predicted ADHD status. While results did not support initial hopes that ex-Gaussian parameters of performance might *better* identify ADHD status than standard indices, these findings support ongoing work in developing and improving process-based approaches in clinical assessment.

## Supplementary Material

Supplementary Material

## Figures and Tables

**Table 1.** Description of groups. Means, with standard deviation in parentheses

	Control	ADHD	Test statistics
**Preschool sample**			
*N* (Boys:Girls)	33 (18:15)	75 (52:23)	*χ*^2^ (1 df) = 219, *p* = .104
Age in years	5.30 (.47)	5.27 (.45)	*F*(1, 106) = .15, *η*^2^ = .001, *p* = .701
Estimated FSIQ	107.27 (8.94)	98.56 (12.86)	*F*(1, 106) = 12.46, *η*^2^ = .105 *p* = .001**
**Inattention**			
Total # of symptoms	.30 (.81)	6.49 (2.12)	*F*(1, 106) = 2.63.16, *η*^2^ = .713, *p* < .001**
Parent Conners *t*-score	43.51 (3.56)	60.77 (11.38)	*F*(1, 106) = 72.28, *η*^2^ = .408, *p* < .001**
Teacher Conners *t*-score	46.64 (3.16)	64.47 (16.54)	*F*(1, 106) = 37.55, *η*^2^ = .262, *p* < .001**
**Hyperactivity/Impulsivity**			
Total # of Symptoms	.39 (.83)	6.87 (2.44)	*F*(1, 106) = 220.13, *η*^2^ = .675, *p* < .001**
Parent Conners *t*-score	46.61 (5.01)	66.03 (11.13)	*F*(1, 106) = 91.82, *η*^2^ = .464, *p* < .001**
Teacher Conners *t*-score	44.21 (1.53)	67.79 (13.27)	*F*(1, 106) = 102.04, *η*^2^ = .490, *p* < .001**
**Comorbidity (DISC-IV)**			
# ODD	1	19	*χ*^2^ (1 df) = 7.56, *p* = .006**
**School-aged Sample**			
N (Boys:Girls)	93 (47:46)	216 (143:73)	*χ*^2^ (1 df) = 6.82, *p* = .007**
Age in years	9.66 (1.30)	9.50 (1.21)	*F*(1,293) = .820, *η*^2^ = .003, *p* = .366
Estimated FSIQ	105.01 (8.54)	103.29 (12.81)	*F*(1, 293) = .800, *η*^2^ = .003, *p* = .372
**Inattention**			
Total # of Symptoms	.50 (.65)	7.94 (1.59)	*F*(1, 293)= 1959.50, *η*^2^ = .870, *p* < .001**
Parent Conners *t*-score	46.02 (4.08	70.75 (11.37)	*F*(1, 293) = 389.69, *η*^2^ = .571, *p* < .001**
Teacher Conners *t*-score	46.20 (4.30)	59.88 (11.34)	*F*(1, 293) = 114.663, *η*^2^ = .281, *p* < .001**
**Hyperactivity/Impulsivity**			
Total # of Symptoms	.23 (.49)	5.70 (2.74)	*F(1, 293)* = *352.760, η*^2^ = *.547, p* < .001**
Parent Conners *t*-score	45.89 (3.62)	68.91 (14.26)	*F(1, 293)* = *223.743, η*^2^ = *.433, p* < .001**
Teacher Conners *t*-score	45.31 (2.96)	59.04 (11.88)	*F*(1, 293) = 115.737, *η*^2^ = .283, *p* < .001**
**Comorbidity (DISC-IV)**			
# MDD/Dysthymia	0/0	10/4	*χ*^2^ (1 df) = 5.344, *p* = .013*
# GAD	1	26	*χ*^2^ (1 df) = 9.25, *p* = .001**
# ODD/CD	3/0	81/20	*χ*^2^ (1 df) = 38.96, *p* < .001**
# SLD	10	19	*χ*^2^ (1 df) = .69, *p* = .41
**Adult sample**			
N (Men:Women)	72 (42:30)	61 (26:35)	*χ*^2^ (1 df) = 3.26, *p* = .051
Age in years	21.19 (.50)	21.13 (1.80)	*F*(1, 131) = .035 *η*^2^ < .001, *p* = .851
Estimated FSIQ	110.32 (11.73)	105.25 (11.08)	*F*(1, 131) = 6.499 *η*^2^ = .047, *p* = .012*
**Inattention**			
Total # of Symptoms	.04 (.26)	6.21 (2.05)	*F(1, 131)* = *640.835 η*^2^ = *.830, p* < .001**
CAARS Inattention *t*-score	46.21 (9.46)	67.39 (15.43)	*F(1, 131)* = *247.873 η*^2^ = *.654, p* < .001**
**Hyperactivity/Impulsivity**			
Total # of symptoms	.10 (.34)	4.61 (2.40)	*F(1, 131)* = *94.092 η*^2^ = *.418, p* < .001**
CAARS hyperactivity	42.25 (8.54)	57.49 (13.76)	*F*(1, 131) = 60.765 *η*^2^ = .317, *p* < .001**

*Note*. MDD, Major Depressive Disorder; GAD, Generalized Anxiety Disorder; ODD, Oppositional Defiant Disorder; CD, Conduct Disorder; SLD, Specific Learning Disability (i.e. WIAT-III standard score <70 on Wording Reading, Spelling, or Numerical Operations); CAARS, Connor’s Adult ADHD Rating Scale. *χ*^2^ analyses reported for comorbidity rates are summed across similar diagnoses (e.g. MDD and Dysthymia; ODD and CD).

**Table 2.** Performance on go-no-go (GNG) and stop signal reaction time (SSRT) tasks by diagnostic group

	Preschool sample GNG task			School-aged sample SSRT task			School-aged sample GNG task			Adult sample GNG task		
	Control	ADHD	Test statistics	Control	ADHD	Test statistics	Control	ADHD	Test statistics	Control	ADHD	Test statistics
SSRT	—	—	—	333.48 (108.23)	441.57 (150.31)	*F*(1,269) = 32.64 *p* < .001** *η*^2^ = .109	—	—	—	—	—	—
% Failed inhibits	15.32 (10.30)	23.04 (18.37)	*F*(1,106) = 5.10 *p* = .026* *η*^2^ = .046	—	—	—	31.60 (14.03)	43.49 (15.90)	*F*(1,104) = 14.06 *p* < .001** *η*^2^ = .120	11.21 (8.55)	21.13 (14.64)	*F*(1,132) = 23.61 *p* < .001** *η*^2^ = .153
RT to “Go” MRT	660.54 (125.83)	711.87 (143.92)	*F*(1,106) = 3.14 *p* = .079 *η*^2^ = .029	791.50 (167.20)	821.46 (169.59)	*F*(1,269) = 1.72 *p* = .191 *η*^2^ = .006	583.59 (85.91)	602.23 (87.37)	*F*(1,104) = 1.07 *p* = .302 *η*^2^ = .01	355.85 (37.81)	348.01 (47.21)	*F*(1,132) = 1.13 *p* = .290 *η*^2^ = .009
SDRT	199.77 (63.11)	242.93 (71.84)	*F*(1,106) = 8.88 *p* = .004** *η*^2^ = .077	209.49 (167.20)	247.95 (80.78)	*F*(1,269) = 13.42 *p* < .001** *η*^2^ = .048	187.51 (35.46)	219.58 (38.75)	*F*(1,104) = 16.90 *p* < .001** *η*^2^ = .141	64.50 (12.51)	73.38 (15.82)	*F*(1,132) = 13.07 *p* < .001** *η*^2^ = .091
mu	486.22 (137.47)	510.11 (170.65)	*F*(1,106) = .50 *p* = .480 *η*^2^ = .005	621.84 (157.35)	624.07 (181.56)	*F*(1,269) = .009 *p* = .925 *η*^2^ <.001	412.81 (68.96)	401.99 (89.07)	*F*(1,104) = .396 *p* = .530 *η*^2^ = .004	299.74 (35.46)	282.52 (41.44)	*F*(1,132) = 6.67 *p* = .011* *η*^2^ = .048
sigma	83.66 (62.32)	108.98 (77.95)	*F*(1,106) = 2.71 *p* = .102 *η*^2^ = .025	111.83 (47.98)	134.12 (72.61)	*F*(1,269) = 6.11 *p* = .014* *η*^2^ = .022	76.21 (27.48)	93.32 (35.58)	*F*(1,104) = 6.23 *p* = .014* *η*^2^ = .057	29.52 (11.21)	31.64 (11.65)	*F*(1,132) = 1.14 *p* = 288. *η*^2^ = .009
tau	174.30 (74.89)	202.04 (96.63)	*F*(1,106) = 2.15 *p* =.146 *η*^2^ = .020	169.66 (96.93)	197.40 (93.78)	*F*(1,269) = 4.69 *p* = .031* *η*^2^ = .017	170.78 (41.31)	200.24 (47.57)	*F*(1,104) = 9.74 *p* = .002** *η*^2^ = .086	56.12 (14.48)	65.49 (16.53)	*F*(1,132) = 12.17 *p* = .001** *η*^2^ = .085

*Note.* Means, with standard deviation in parentheses. MRT = mean reaction time. SDRT = standard deviation of reaction time.

* *p* < .05

** *p* < .01.

**Table 3.** Area under the curve (AUC) statistics by sample and task parameter

	Preschool sample GNG task			School-aged sample SSRT task			School-aged sample GNG task			Adult sample GNG task		
	AUC	Std. Error	Asymp. Sig	AUC	Std. Error	Asymp. Sig	AUC	Std. Error	Asymp. Sig	AUC	Std. Error	Asymp. Sig
SSRT	—	—	—	.717	.033	<.001***	—	—	—	—	—	—
% Failed	.608	.054	.076	—	—	—	.708	.053	.001**	.730	.044	<.001**
Inhibits												
RT to “go”												
MRT	.605	.058	.084	.569	.04	.078	.556	.060	.352	.418	.050	.103
SDRT	.671	.055	.005**	.659	.037	<.001***	.727	.051	<.001**	.666	.047	.001***
mu	.543	.060	.482	.498	.041	.956	.432	.059	.256	.334	.048	.001***
sigma	.581	.060	.179	.592	.039	.018**	.694	.056	.001**	.544	.051	.383
tau	.606	.056	.079	.593	.038	.018**	.691	.055	.002**	.655	.047	.002**

Note.

** *p* < .01

*** *p* < .001.

**Table 4.** Comparing diagnostic discriminability across the most consistently performing parameters

	Preschool sample GNG task				School-aged sample SSRT task				School-aged sample GNG task				Adult sample GNG task			
	*r*_Control_	*r*_ADHD_	*z*	*p*	*r*_Control_	*r*_ADHD_	*z*	*p*	*r*_Control_	*r*_ADHD_	*z*	*p*	*r*_Control_	*r*_ADHD_	*z*	*p*
SSRT *vs.* SDRT	—	—	—	—	.21	.337	1.34	.18	—	—	—	—	—	—	—	—
SSRT *vs.* sigma	—	—	—	—	−.129	−.082	2.64	.01**	—	—	—	—	—	—	—	—
SSRT *vs.* tau	—	—	—	—	.258	.476	3.03	.003**	—	—	—	—	—	—	—	—
SDRT *vs.* tau	.721	.616	1.38	.17	.836	.65	2.37	.02*	.929	.804	1.19	.23	.731	.937	.37	.71
%FI *vs.* SDRT	−.028	.217	−.85	.40	—	—	—	—	.113	.212	−.28	.78	.105	−.109	.104	.30
%FI *vs.* sigma	−.131	.001	.34	.73	—	—	—	—	.028	.098	.19	.85	.007	−.283	2.96	.003**
%FI *vs.* tau	.118	.209	.03	.97	—	—	—	—	.041	.127	.23	.82	.008	−.092	.119	.23

*Note.* %FI, % failed inhibits; *r*, Pearson’s correlation between paired AUC values; by ADHD status. *z* = critical *z* value of the observed difference between AUC of two parameters.

* *p* < .05

** *p* < .01. A logistic regression performed to evaluate the effects of SSRT, MRT, SDRT, mu, sigma, and tau together in the prediction of ADHD status in the school-aged sample supported existing results. Ex-Gaussian parameters continued to be outperformed by SSRT in the prediction of ADHD status. Specifically, the logistic regression model was statistically significant, *χ*^2^(6) = 48.01, *p* < .001, Nagelkerke, *R*^2^ = .23. However, only SSRT predicted ADHD status, Wald *χ*^2^ = 19.13, *p* < .001 (all other *χ*^2^ < 2.2, all *p* > .14).

## Footnotes

1 Average correct inhibit rate were in the expected range for both non-ADHD controls = 52% and children with ADHD = 56%. Eleven children (all ADHD) had < 20% correct inhibits; the remaining participants were within range (20–58%). Removing these n = 11 children did not change results, so results are reported with those children included.
