# Supplementary material for: Ahead of the (ROC) Curve: A Statistical Approach to Utilizing Ex-Gaussian Parameters of Reaction Time in Diagnosing ADHD Across Three Developmental Periods
Source: J Int Neuropsychol Soc. Author manuscript; Available in PMC 2023 Sep 1. (PMC9521363; doi:10.1017/S1355617721000990)
Supplement: Supplementary Material [file NIHMS1780514-supplement-Supplementary_Material.docx]

**Supplemental Table 1.** Area Under the Curve (AUC) Statistics by sample, gender, and task parameter. Primary results and interpretation did not vary by gender.

|  |  | **Preschool GNG Task** | | | **School-aged SSRT** | | | **School-aged GNG** | | | **Adult GNG** | | |
| --- | --- | --- | --- | --- | --- | --- | --- | --- | --- | --- | --- | --- | --- |
|  |  | AUC | Std. Error | Asymp. Sig | AUC | Std. Error | Asymp. Sig | AUC | Std. Error | Asymp. Sig | AUC | Std. Error | Asymp. Sig |
| Boys | SSRT | -- | -- | -- | 0.76 | 0.04 | <.001 | -- | -- | -- | -- | -- | -- |
|  | % Failed inhibits | 0.49 | 0.07 | 0.94 | -- | -- | -- | 0.69 | 0.07 | 0.02 | 0.73 | 0.06 | 0.00 |
|  | MRT | 0.74 | 0.07 | 0.00 | 0.55 | 0.06 | 0.36 | 0.58 | 0.08 | 0.31 | 0.46 | 0.07 | 0.62 |
|  | SDRT | 0.73 | 0.07 | 0.00 | 0.71 | 0.05 | <.001 | 0.72 | 0.07 | 0.01 | 0.70 | 0.07 | 0.01 |
|  | mu | 0.66 | 0.08 | 0.04 | 0.45 | 0.06 | 0.32 | 0.44 | 0.08 | 0.46 | 0.38 | 0.07 | 0.10 |
|  | sigma | 0.64 | 0.08 | 0.08 | 0.54 | 0.06 | 0.52 | 0.67 | 0.08 | 0.03 | 0.62 | 0.07 | 0.11 |
|  | tau | 0.64 | 0.07 | 0.08 | 0.67 | 0.05 | 0.00 | 0.73 | 0.07 | 0.00 | 0.69 | 0.07 | 0.01 |
| Girls | SSRT | -- | -- | -- | 0.69 | 0.05 | 0.00 | -- | -- | -- | -- | -- | -- |
|  | % Failed Inhibits | 0.75 | 0.08 | 0.01 | -- | -- | -- | 0.68 | 0.09 | 0.06 | 0.73 | 0.06 | 0.00 |
|  | MRT | 0.51 | 0.10 | 0.89 | 0.62 | 0.06 | 0.04 | 0.58 | 0.09 | 0.42 | 0.39 | 0.07 | 0.12 |
|  | SDRT | 0.63 | 0.09 | 0.17 | 0.66 | 0.05 | 0.00 | 0.76 | 0.08 | 0.01 | 0.63 | 0.07 | 0.07 |
|  | mu | 0.42 | 0.09 | 0.41 | 0.55 | 0.06 | 0.38 | 0.46 | 0.09 | 0.64 | 0.30 | 0.07 | 0.01 |
|  | sigma | 0.52 | 0.10 | 0.83 | 0.64 | 0.06 | 0.02 | 0.73 | 0.08 | 0.02 | 0.48 | 0.07 | 0.74 |
|  | tau | 0.57 | 0.10 | 0.48 | 0.59 | 0.06 | 0.13 | 0.70 | 0.08 | 0.04 | 0.61 | 0.07 | 0.13 |

**Supplemental Table 2.** Comparing diagnostic discriminability across the most consistently performing parameters by Gender. Primary results and interpretation did not vary by gender.

|  |  | **Preschool GNG** | | | | | **School Aged SSRT** | | | | | **School Aged GNG** | | | | | **Adult GNG** | | | |
| --- | --- | --- | --- | --- | --- | --- | --- | --- | --- | --- | --- | --- | --- | --- | --- | --- | --- | --- | --- | --- |
|  | | r Control | r ADHD | z | *p* | r Control | | r ADHD | z | *p* | r Control | | r ADHD | z | *p* | r Control | | r ADHD | z | *p* |
| **Boys** | |  |  |  |  |  | |  |  |  |  | |  |  |  |  | |  |  |  |
| SSRT vs SDRT | | -- | -- | -- | -- | 0.14 | | 0.34 | 0.78 | 0.44 | -- | | -- | -- | -- | -- | | -- | -- | -- |
| SSRT vs. sig | | -- | -- | -- | -- | -0.04 | | -0.09 | 3.23 | 0.001 | -- | | -- | -- | -- | -- | | -- | -- | -- |
| SSRT vs tau | | -- | -- | -- | -- | 0.19 | | 0.53 | 1.63 | 0.10 | -- | | -- | -- | -- | -- | | -- | -- | -- |
| SDRT vs tau | | 0.69 | 0.73 | 1.57 | 0.12 | 0.73 | | 0.53 | -2.39 | 0.02 | 0.91 | | 0.96 | -0.20 | 0.84 | 0.56 | | 0.94 | 0.14 | 0.89 |
| %FI vs SDRT | | 0.14 | 0.35 | -2.76 | 0.01 | -- | | -- | -- | -- | 0.20 | | 0.21 | -0.31 | 0.76 | 0.07 | | -0.02 | 0.32 | 0.75 |
| %FI vs sig | | -0.03 | 0.18 | -1.43 | 0.15 | -- | | -- | -- | -- | -0.09 | | 0.08 | 0.19 | 0.85 | 0.05 | | -0.03 | 1.19 | 0.23 |
| %FI vs tau | | 0.27 | 0.23 | -1.73 | 0.08 | -- | | -- | -- | -- | 0.15 | | 0.13 | -0.37 | 0.71 | -0.12 | | -0.10 | 0.41 | 0.68 |
| **Girls** | |  |  |  |  |  | |  |  |  |  | |  |  |  |  | |  |  |  |
| SSRT vs SDRT | | -- | -- | -- | -- | 0.21 | | 0.37 | 0.37 | 0.71 | -- | | -- | -- | -- | -- | | -- | -- | -- |
| SSRT vs. sig | | -- | -- | -- | -- | -0.15 | | -0.07 | 0.71 | 0.48 | -- | | -- | -- | -- | -- | | -- | -- | -- |
| SSRT vs tau | | -- | -- | -- | -- | 0.25 | | 0.43 | 1.58 | 0.11 | -- | | -- | -- | -- | -- | | -- | -- | -- |
| SDRT vs tau | | 0.73 | 0.40 | 0.69 | 0.49 | 0.87 | | 0.82 | 1.53 | 0.13 | 0.95 | | 0.48 | 0.90 | 0.37 | 0.95 | | 0.93 | 0.58 | 0.56 |
| %FI vs SDRT | | 0.06 | 0.00 | 0.94 | 0.35 | -- | | -- | -- | -- | 0.06 | | 0.37 | -0.77 | 0.44 | 0.17 | | -0.15 | 1.10 | 0.27 |
| %FI vs sig | | -0.16 | -0.21 | 0.23 | 0.82 | -- | | -- | -- | -- | 0.21 | | 0.19 | -0.46 | 0.65 | -0.12 | | -0.42 | 3.04 | 0.002 |
| %FI vs tau | | 0.18 | 0.20 | 1.53 | 0.12 | -- | | -- | -- | -- | -0.02 | | 0.17 | -0.17 | 0.86 | 0.22 | | -0.09 | 1.32 | 0.19 |
